# Complete resolution of obstructive colonic amebic pseudotumor with conservative treatment: A case report and literature review

**DOI:** 10.1016/j.ijscr.2019.04.046

**Published:** 2019-05-07

**Authors:** Elias Chahine, Ramez Baghdady, Claude Chahine, Ghassan Doghman, Nader EL Kary, Lionel El Khoury, Kassem Safa, Marc-Anthony Chouillard, Elie Chouillard

**Affiliations:** a Department of Minimally Invasive Surgery, Poissy Saint Germain Medical Center, Poissy, France; b Department of Hematology, Gustave Roussy Institute, University Paris-Saclay, Villejuif, France; c Department of General Surgery, El Zahraa University Hospital Beirut Lebanon, Lebanon; d Transplant Center and Division of Nephrology, Massachusetts General Hospital, Harvard Medical School, Boston, MA, USA

**Keywords:** *Entamoeba histolytica*, Infectious colitis, Amoeboma, Amoebic colitis, Tumor, Case report

## Abstract

**Introduction:**

Entamoeba histolytica is a well-known cause of infectious colitis, it has a worldwide distribution. Presentation ranges from mild diarrhea to occasionally frank dysentery and may spread to involve extra intestinal sites such as the liver, lung, and other organs, in the form of amebic abscesses.

**Case presentation:**

Herein, we report a case of amebic pseudotumor wherein the diagnosis was revealed after 2 months of diarrhea.

**Discussion:**

Colonic amebiasis, may rarely form a segmental mass called amebic pseudotumor in patient untreated or inadequately treated during the course of proven amebic colitis.

Due to the rarity of colonic amoebic pseudo tumor it is usually discovered incidentally during surgical interventions.

However if discovered earlier it responds well to medical treatment and usually resolves within few weeks.

**Conclusion:**

So surgical intervention is reserved for complications of this entity manifested by fulminant colitis or colonic perforation.

## Highlights

• Amebomas are an uncommon complication of invasive amebiasis.
• If a typical clinical picture of amebic colitis, diagnosis may be difficult.
• A luminal agent may be used to eradicate carriage post amebiasis treatment.
• Amebomas respond well to high-dose metronidazole.

## 1 Introduction

*Entamoeba histolytica* is a well-known cause of infectious colitis, and causes 34–50 million symptomatic infections annually worldwide, resulting in 40,000–100,000 deaths each year. Despite its global distribution, it is more common in developing countries with populations at risk in widespread regions, particularly in the tropics and subtropics. Its presentation ranges from mild diarrhea to occasionally frank dysentery and may spread to involve extra intestinal sites such as the liver, lung, and other organs, usually in the form of amebic abscesses, particularly liver abscesses. Years after the last episode of dysentery, localized infection of the colon may, rarely, form a segmental mass, called an “ameboma,” in patients untreated or inadequately treated during the course of proven amebic colitis. The diagnosis of amebomas is difficult if the clinical picture of amebic colitis is unclear. We present a rare case of amebic pseudotumor wherein the diagnosis was revealed after 2 months of diarrhea.

The work in this case has been reported in line with the SCARE criteria [1].

## 2 Presentation of case

A 51-year-old male patient presented with acute abdominal discomfort with postprandial abdominal distention, associated with diarrhea of 2 months duration. He had no weight loss, melena, loss of appetite, and no other gastrointestinal symptoms. His family history was unremarkable. Physical examination revealed slight tenderness in the right iliac fossa, where a large mass was palpable. The result of the blood tests including complete blood count, electrolytes, BUN, creatinine, liver function tests, amylase and lipase were unremarkable; biochemical parameters including tumor markers (Ca19.9: 20 U/ml (nl <37 U/ml) and ACE: 1 ng/ml (<5 ng/ml)) were within the normal limits. Parasitology and bacteriology stool examinations showed normal findings.

**Fig. 1:**
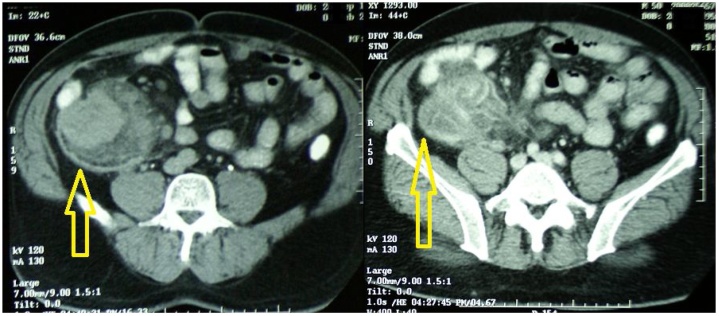
CT scan of the abdomen: (arrows) shows the pseudotumor.

A computed tomography scan of the abdomen showed marked concentric wall thickening of the terminal ileum and cecum, and an ascending colon tumor measuring 8 × 8 cm, with lumenal narrowing at the level of the hepatic flexure (Fig. 1). The tumor showed central low-density content, suggestive of an inflammatory etiology. A colonoscopy showed obstruction at the hepatic flexure; the colonoscope could not traverse the obstruction due to severe lumenal narrowing. However, no notable findings were observed distally to the defined lesion. Histological examination of a biopsy specimen taken from the edematous mucosa by colonoscopy revealed inflammatory cell infiltration without ulceration.

While awaiting results and with a high index of suspicion of an amebic pseudotumor, treatment was initiated with metronidazole (1500 mg/day). Three days after initiation of treatment, the symptoms disappeared; repeated colonoscopy following the improvement of symptoms showed normal large bowel up to 12 cm in the ileum. The treatment was continued for 2 weeks and a repeated abdominal scan showed total disappearance of the tumor.

## 3 Discussion

Amebiasis is caused by a protozoan, and is a major cause of morbidity. Worldwide, it is the third leading cause of death due to parasitic diseases in humans, after malaria and schistosomiasis. Amebiasis is the infection of the human gastrointestinal tract by *Entamoeba histolytica.* It was first seen in 1878 but first described in 1903. *E. histolytica* is a unicellular protozoan organism, which is characterized by motility via pseudopod extension. Seven protozoan species in the genus *Entamoeba* infect humans, but not all of them are pathogenic. *E. histolytica* is the primary pathogen; non-pathogenic species (e.g., *E. dispar*, *E. coli*, *E. moshkovskii*, and *E. hartmanii* [2]) are important because they are morphologically indistinguishable from *E. histolytica* and may be confused with *E. histolytica* during diagnostic evaluation [3].

While endemic in large regions of the tropics, amebiasis also occurs sporadically in the temperate climates of developed countries, most commonly in immigrants, travelers, refugees, aboriginals, residents of penal and mental institutions, and in male homosexuals. Humans are the only known host of the parasite *E. histolytica*. Transmission of *E. histolytica* is by the fecal-oral route. Infection starts with ingestion of the cyst of *E. histolytica* from fecally contaminated food or water. The cysts are susceptible to heat (temperature >40 °C), freezing (temperature <−5 °C), and drying and remain viable in a moist environment for 1 month [3]. The cyst excysts in the small intestine (ileocecal valve) in an alkaline environment to form a total of eight trophozoites that move down into the large intestine, which then multiply by binary fission and subsequently form cysts that are excreted in the feces to start a new cycle. The trophozoites colonize the large intestine and invade the mucosa; they live within the crypts and mucosa of the large intestinal lining and may multiply indefinitely within the crypts of the large intestine mucosa feeding on starches and mucous secretions [3].

Rarely, patients with long-standing *E. histolytica* infection develop tumorous, exophytic, cicatricial, and inflammatory masses known as “amebomas’’ as an uncommon complication of invasive amebiasis. Amebomas result from the formation of annular colonic granulation tissue at single or multiple sites, usually within the cecum or ascending colon [4]. The tissue necrosis that characterizes amebic colitis is replaced by an extensive inflammatory reaction and pseudotumor formation in ameboma, possibly because of secondary bacterial infection. It occurs in 1.5% of all cases of invasive amebiasis. Ameboma usually occurs in untreated or inadequately treated patients with amebiasis years after the last episode of dysentery. Amebomas are usually solitary, of variable size, and can be up to 15 cm in diameter. Men between 20–60 years of age are most commonly affected. In decreasing order of frequency, lesions develop in the cecum, the appendix, and the rectosigmoid region [4]. Other sites include the hepatic flexure, transverse colon, and splenic flexure.

Amebomas may cause obstructive symptoms. Alternating diarrhea and constipation, weight loss, and low-grade fever may be seen. In endemic areas, cramping lower abdominal pain and a palpable mass suggest the diagnosis. The differential diagnoses include Crohn’s disease and appendiceal abscesses in younger individuals, and colon cancer and diverticulitis in the elderly.

Rarely, colonic ameboma may be accompanied by amebic liver abscesses and may be misdiagnosed as metastatic carcinoma of the colon. Endoscopic evaluation yields a definitive diagnosis in about 66% of cases; biopsy specimens may be required to distinguish the lesion from carcinoma or a large adenoma [5,6].

Because cecal ameboma is a rare condition, it is often discovered only at laparotomy. It responds well to pharmacologic therapy and usually disappears within a few weeks after treatment. Surgical intervention may be required if complications such as fulminant amebic colitis and colonic perforation occur.

In the absence of the clinical picture of amebic colitis, as in the present case, diagnosis may be difficult. Only a few cases of ameboma have been reported to date. In all reported cases, cecal ameboma was diagnosed when surgery was performed for a preliminary diagnosis of acute appendicitis, carcinoma, or lymphoma, or after histopathologic evaluation of a colonic mass discovered unexpectedly during surgery [7,8].

## 4 Conclusion

In summary, colonic amoeboma is a rare complication of invasive amoebiasis and the management of this amebic pseudotumor is a rather surgical or intravenous antiparasitic agents. The equivocal clinical symptoms that differentiate invasive amebiasis from Crohn disease and even colon cancer may be controversial.

Discerning amebic pseudotumor from other pathology by endoscopy with biopsy and serology bring crucial information. Since most surgeons are probably to confront few case, recognition of this pathology is a must.

## Conflicts of interest

No potential conflict of interest relevant to this article was reported.

## Funding

There are no sources of funding for this research.

## Ethical approval

Not applicable. The study is exempt from ethical approval in our institution.

## Consent

Consent has been obtained from the patient. No identifying details have been used in the article.

## Author contribution

E CHOUILLARD: study concept, and final approval. G Doghman and E Chahine treated the patient. E Chahine and R Baghdady wrote the first draft of the manuscript. L El khoury, N El kary, and C Chahine wrote the final draft of the manuscript, MA Chouillard photo editing, K SAFA in editing manuscript. All authors read and approved the final manuscript.

## Registration of research studies

Not available.

## Guarantor

CHAHINE E.

## Provenance and peer review

Not commissioned, externally peer-reviewed.
